# Evaluation of shear bond strength of zirconia to composite resin using different adhesive systems

**DOI:** 10.4317/jced.55428

**Published:** 2019-03-01

**Authors:** Faramarz Zakavi, Mehrnoosh Mombeini, Sana Dibazar, Sarah Gholizadeh

**Affiliations:** 1 Assistant Professor, Operative and Esthetic Dentistry ,Dental Faculty, Ahvaz Jundishapur Medical Science University, Ahvaz, Iran; 2 Assistant Professor, Operative and Esthetic Dentistry, Dental Faculty, Khorram Abad University of Medical science, Khorram Abad, Iran; 3 Post Graduate Student, Operative and Esthetic Dentistry, Dental Faculty, Ahvaz Jundishapur Medical Science University, Ahvaz, Iran; 4 Assistant Professor, Operative and Esthetic Dentistry, Dental Faculty, Ahvaz Jundishapur Medical Science University, Ahvaz, Iran

## Abstract

**Background:**

To evaluate shear bond strength of zirconia to composite resin using different universal and conventional adhesives and a zirconia primer.

**Material and Methods:**

Forty zirconia blocks were fabricated of zirconium ingots (10×10×5 mm) and sintered at 1530°C for 2 hours. They were then air-abraded with Al2O3 particles. The specimens were divided into 4 groups and subjected to one of the following bonding agents: Futurabond U (group 1), Clearfil Universal Bond, universal adhesives (group 2), Z-Prime Plus, zirconia primer (group 3) and Adper Single Bond 2, conventional adhesive (group 4). Composite resin was then applied in a diameter of 5 mm and in a thickness of 2 mm. All the specimens were stored in distilled water at 37°C for 24 hours and then thermocycled between 5°C and 55°C for 5000 cycles with a 30-second dwell time. The shear bond strength was then evaluated with a universal testing machine at a crosshead speed of 1 mm/min. Data (MPa) were analyzed using ANOVA and LSD test (*P*≤0.05). The specimens were evaluated under a stereomicroscope to determine the mode of failure.

**Results:**

The mean shear bond strength was 16,874 MPa in group I, 13.4434 MPa in group II, 11.6500 MPa in group III and 6.8700 MPa in group IV. ANOVA revealed that the shear bond strength in group IV was significantly lower than that in other groups (*P*≤0.05).

**Conclusions:**

The shear bond strength in group I was significantly higher than that in groups III and IV. So Universal adhesives could provide higher shear bond strength of zirconia to composite resin after thermocycling compared to zirconia primers.

** Key words:**10-MDP, shear bond strength, universal adhesive systems, zirconia primer.

## Introduction

Use of zirconia is on the increase in modern dentistry due to its superior biocompatibility and favorable mechanical properties (1). Zirconia is usually used as a core for restorations due to the high opacity of zirconia ceramic so that a proper esthetic appearance can be achieved; subsequently the core is veneered with the use of feldspathic ceramic (2).

A large number of studies have confirmed the clinical success of restorations with the use of zirconia cores. However, the zirconia core‒feldspathic veneer interface has been reported as one of the chief weak points of these restorations, which might result in the exposure of the underlying zirconia (3-6). In such cases, whether the restoration should be repaired or replaced depends on the location and the extent of chipping of the feldspathic veneer. The repair of a defective restoration is advantageous to its replacement due to the lower cost of the repair process and the possibility of the repair in one visit (7).

Feldspathic ceramics are etched with the use of hydrofluoric acid and the chemical bond to composite resin improves by applying silane to the etched surface (8). However, since the structure of zirconia is crystalline, with no glass, etching with hydrofluoric acid is not effective in preparing its surface. On the other hand, a deficiency of silica in its structure does not allow adequate bonding of composite resin (9). Therefore, techniques such as grinding, air abrasion with aluminum oxide particles and tribochemical silicoating, selective etching, etching of the surface with laser beams and application of adhesive/primer of zirconia are used to this end (10).

The results of the use of primers containing 10-MPD (10-methacryloyloxydecyl dihydrogen phosphate) for improving the adhesion of zirconia to composite resin have been promising (9,11,12). This monomer has two functional groups: one phosphate group which is responsible for bonding to the hydroxyl group on the zirconia surface and one carboxylic acid group which is light-cured and is bonded to composite resin (11,13,14). Despite the amphiphilic property mentioned for this monomer, it is still the most hydrophobic functional monomer among the dental adhesive/primers, which results in better durability of the bond in the oral cavity. Based on previous studies, Z-Prime Plus primer is the most effective among primers containing MDP (10,15,16,17). This product, which was marketed in 2009 for use as a zirconia primer, is a specific composition of MDP hydrophilic monomers, HEMA, carboxylic monomer and Bis-GMA hydrophobic resin monomer, dissolved in water and ethanol (18,19).

Universal adhesives are a new group of adhesives that, based on manufacturer’s claim, have the capacity to be used with all the techniques, including total etch, selective and self-etch and can bond to all the direct substrates such as enamel and dentin and indirect substrates such as metals, zirconia and glass ceramics, in one product. These adhesives, too, contain MDP monomer, which explains their etching capacity in association with their bond strength to the substrates mentioned above (16).

The ideal technique for preparation of zirconia to create an effective chemomechanical bond to composite resin is still unknown (20). Considering the diversity of commercial products available, including primer/adhesives, there is still a lack of clear and uniform guideline for bonding of zirconia to composite resin. The aim of the present study was to evaluate the effect of one zirconia primer, Z-Prime, and two universal adhesives, Clearfil Universal Futura Bond U (containing MDP) and Adper Single Bond 2 (without MDP monomer and only for evaluating the effect of wetting) on the shear bond strength of composite resin to zirconia under the same surface preparation conditions.

## Material and Methods

This *in-vitro* study was conducted on 40 YTZP zirconia discs to assess their shear bond strength to composite resin using different adhesives. The zirconia blocks (Vita In-Ceram YZ, Vita Zahnfabrik, Bad Säckingen, Germany) were cut with a diamond cutting blade (3 Axes Full Automatic; Nemo Fanavaran Pars, Mashhad, Iran) to fabricate discs measuring 10×10×5 mm. The samples were polished using silicon carbide paper discs up to 1000-grit under water cooling to achieve standardized surfaces. After 15 seconds of rinsing with distilled water, the discs were then sintered according to the manufacturer’s instructions at 1530°C for 2 hours (LHT 02/16, Nabertherm GmbH, Lilienthal/Bremen, Germany).

The samples were sandblasted for 15 seconds using 50-µm aluminum oxide particles at a 10-mm distance and 0.25 MPa pressure perpendicular to the surface using a sandblaster (Microetcher II, Danville Engineering Inc, San Ramon, California, USA ) and a Teflon strip with a 5-mm hole to outline the bonding area. To apply the adhesive, the samples were classified into 4 groups as follows (the manufacturers’ instructions were followed in each group).

Group 1: Futrabond U adhesive (Voco GmbH, Cuxhaven, Germany ), a single-dose adhesive consisting of two components. After mixing the components, the adhesive was applied on the surface with a micro-brush, thinned for 5 seconds using air spray and then cured for 10 seconds.

Group 2: Clearfil universal bond adhesive (Kuraray Noritake Denta Inc., Okayama, Japan). First, the bonding surface was etched using 40% phosphoric acid etchant (K-Etchant Syringe, Kuraray) for 5 seconds. Then, the surface was rinsed and dried, and one layer of Clearfil Universal was applied with a micro-brush and rubbed on the surface for 10 seconds. Then, gentle air spray was used for 5 seconds and the bonding agent was cured for 10 seconds.

Group 3: Z-Prime Plus primer (BISCO, Schaumburg, IL, USA). A thin layer of Z-Prime was applied on the bonding surface with a micro-brush and allowed to dry. The second layer was applied as such and gentle air spray was used for 3‒5 seconds. It was then cured for 10 seconds.

Group 4: Adper Single Bond 2 adhesive (3M ESPE, Saint Paul, MN, USA). The bonding surface was etched using 40% phosphoric acid (K-Etchant Syringe, Kuraray) for 15 seconds, rinsed for 10 seconds and dried for 5 seconds using air spray. Then, a thin layer of Adper Single Bond 2 was applied with a micro-brush and thinned with air spray. The second layer was applied as such and cured for 10 seconds.

In each group, the adhesive was applied according to the manufacturers’ instructions and all were light-cured using an LED light-curing unit (Nichia Chemical Industries, 2000 mcd, Japan) with a light intensity of 1200 mW/cm2.

In the next step, A3 shade of Valux Plus composite resin (3M ESPE, St Paul, MN, USA) was applied on each sample by using Tygon tubes (Saint Gobain, Akron, OH, USA) with a height of 2 mm and an internal diameter of 5 mm (to standardize composite resin size) and light-cured for 40 seconds. After one hour, the Tygon tubes were cut with a scalpel blade and removed.  Table 1 shows the materials used in this study.

**Table 1:**
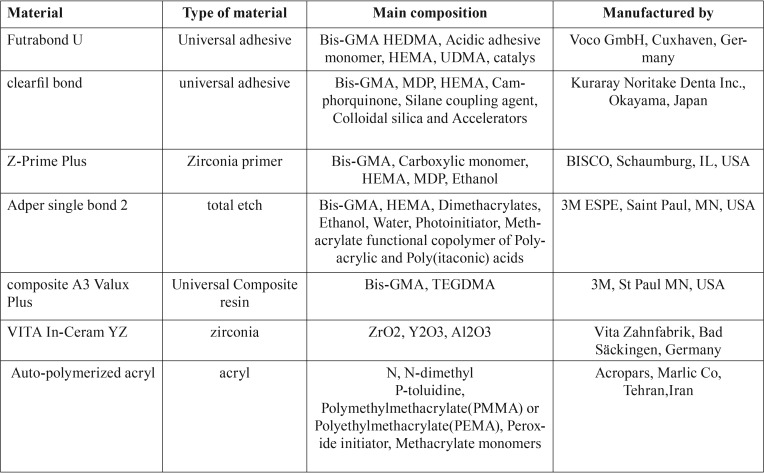
List of materials used in this study and main compositions.

For thermocycling, first the samples were placed in 37°C distilled water at room temperature for 24 hours and then immersed in distilled water at 5‒55°C with a dwell time of 30 seconds for 5000 cycles (TC/300; Vafaei Industrial, Tehran, Iran).

For shear bond strength testing, the samples were mounted in auto-polymerizing acrylic resin (Acropars, Marlic Co, Tehran, Iran) blocks so that the composite‒zirconia interface was 2 mm higher than the acrylic surface. A universal testing machine (Zwick 1445; Zwick, Ulm, Germany) was used for evaluating macroshear bond strength. The mounted samples were fixed to the arms of the machine and the chisel blade applied force parallel to the interface at a crosshead speed of 1 mm/min. Data were reported in MPa.

After bond strength testing, The failure modes were determined for each sample using a stereomicroscope (Motic SMZ-143 SERIES, Micro-optic industrial group Co, Xiamen, China) at 40× magnification. The fracture mechanism was classified into three different types, adhesive failure occured along the adhesive interface, mixed failure occured within the adhesive joint with failure within the resin composite or zirconia, or a cohesive failure which occured within the resin composite or zirconia. Fractures occured during the thermal aging process were considered as prefailure.

Data were analyzed using ANOVA and LSD test for pairwise comparison of data. Statistical analyses were carried out using SPSS 23.

## Results

 Table 2 shows the means, standard deviations, maximums and minimums of shear bond strength values in the groups (MPa).

**Table 2:**
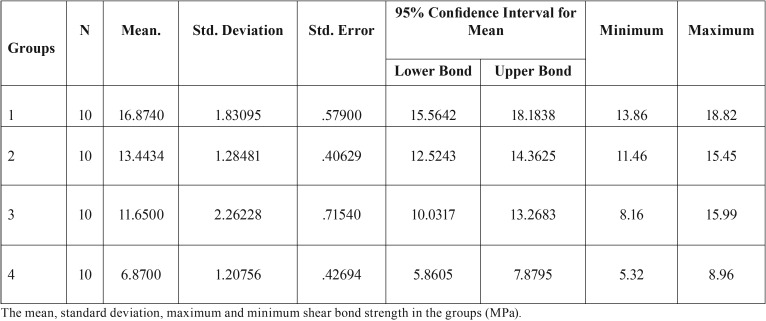
Shear bond strength.

The mean shear bond strength was 16.874 MPa in group 1 (Voco), 13.4434 MPa in group 2 (Clearfil Universal), 11.6500 MPa in group 3 (Z-Prime) and 6.8700 MPa in group 4 (Adper Single Bond 2).

The LSD test was used for pairwise comparisons of data ( Table 3).

**Table 3:**
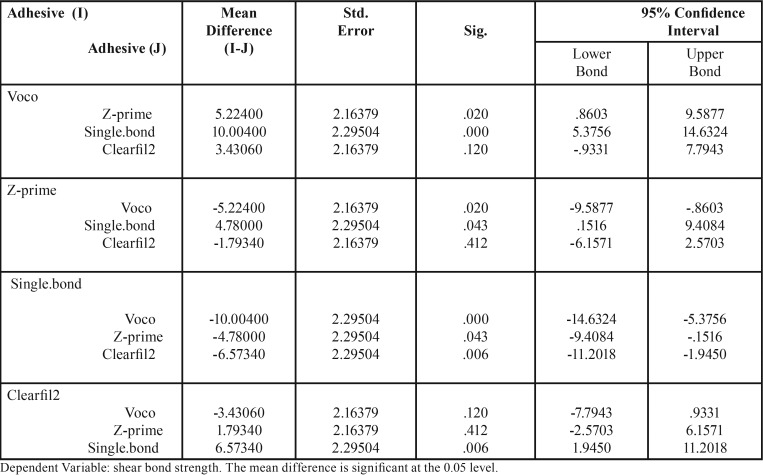
LSD test- pairwise comparison of the data.

The bond strength in group 4 (Single Bond 2) was significantly lower than that in other groups (*P*≤0.05). The bond strength in group 1 was higher than that in group 2 but the difference was not statistically significant (*P*>0.05).

The bond strength in group 1 was significantly higher than that in groups 3 and 4 (*P*≤0.05).

The bond strength in group 2 was higher than that in groups 3 and 4, which was significantly different from group 4 (*P*≤ 0.05).

Failure mode analysis for spicements revealed predominance of mixed failures for Futrabond U , Clearfil universal and Z-Prime Plus.

All samples prepared with Adper Single Bond 2 were classified as adhesive failure and two were fractured before the shear test.

## Discussion

The zirconia‒feldspathic veneer interface is one of the major weak points of these restorations and in some cases results in factures, exposing the underlying zirconia (4-6). Intraoral repair of these restorations is carried out with the use of composite resin to extend their clinical service (21).

In recent years, the results of various studies have indicated the possibility of chemical bonding of functional monomers such as 10-MDP (also present in the composition of universal adhesives) to zirconia (9,11,12,22).

In the present study the effect of one commonly used zirconia primer and two universal adhesives containing 10-MDP monomer and one bonding system without this monomer (as a control) was evaluated on the shear bond strength of composite resin to zirconia under the same surface preparation conditions. In addition, an initial surface preparation was carried out with air abrasion using aluminum oxide particles in order to clean the surface, eliminate impurities, increase surface roughness, change surface energy and increase wettability. This procedure also results in better penetration of resin into surface irregularities and formation of micromechanical interlocking (12,23). Air abrasion without a primer can result in a high bond strength initially but the bond strength decreases steeply over time, even reaching zero (24). In a study on different surface preparation techniques to zirconia although the surface roughness was higher with the use of Nd:YAG laser compared to other groups, surface preparation with air abrasion yielded in higher shear bond strength (25). The shear bond strength decreased significantly after 5000 rounds of thermocycling. Pressure, time and the size of the air abrading particles had no significant effect on the bond strength before thermocycling; however, they clearly resulted in the preservation of the long-term bond strength after thermocycling (12,26).

In this *in-vitro* study, the highest shear bond strength of resin to zirconia was related to Futura Bond U adhesive but this superiority was not significant in comparison to Clearfil Universal Bond. In recent years, 10-MDP monomer has been suggested in association with carboxylic acid, silane and other resin monomers to improve the chemical bond of resin zirconia (9,11,12,27). The hydroxyl group on the zirconia surface can react with the phosphate groups of 10-MDP in the primer. This reaction can lead to the formation of Zr-O-P chemical bond between zirconia ceramic and 10-MDP monomer (11,13,27). A large reaction surface, a higher concentration of functional monomers and a close contact between the two reacting components have roles in forming Zr-O-P bond and improving the bond of zirconia to resin (14,28). One of the differences of the Futura Bond U adhesive from other adhesives containing 10-MDP is the altered chemical structure of the phosphate ester monomer. Although 10-MDP-based monomer is the most hydrophobic functional monomer used in dental adhesives (16), similar to other methacrylate derivatives its esteric bond is susceptible to hydrolysis (21,22). This property might be important in relation to the durability of the bond because water sorption and hydrolytic degradation of the adhesive interface over time are considered primary reasons for the failure of the bond (16). Considering the superiority of the Futura Bond U bond, it is possible that the change in the nature of the monomer has been implemented to increase its resistance to hydrolysis.

Another difference of Futura Bond U from Clearfil Bond Universal adhesive is the fact that Futura Bond U is devoid of silane and contains functional carboxylic ester. The absence of silane in the composition of Futura Bond U makes it possible to increase the concentration of 10-MDP monomer (16,29,30). In addition, silane can form a network structure in the polymerized adhesive, in which water and ethanol are trapped and are eliminated with some difficulty; however, the absence of silane in Futura Bond U adhesive decreases the hydrophobicity of the polymerized adhesive, making it less susceptible to hydrolytic degradation. Besides, elimination of the solvent in Clearfil Bond U adhesive is more difficult due to its higher viscosity. Elimination of water, ethanol and other products that remain in the cavities of silane made network, increases the number of locations available for reaction with 10-MDP, resulting in formation of a better Zr-O-P bond (31), but might be a reason for higher technique sensitivity of Clearfil Bond Universal adhesive.

The poorest results in the present study were achieved with Adper Single Bond 2 adhesive. After thermocycling two samples exhibited bond failure before testing the bond strength. Not involving these prefailures in statistical analysis would overrate the mean results of bond strength, besides assuming 0 MPa would alleviate the mean results vice versa. So in this study the lowest measured value for shear bond strength within the concerned group is assigned for the two prefailed samples (32). The bond between Adper Single Bond 2 and Zirconia is achieved only through micromechanical interlocking of the resin in the surface irregularities of sandblasted zirconia, which might explain the lower bond strength (16).

Another adhesive primer used in the present study was Z-Prime Plus, with a lower bond strength compared to Futura Bond U and a higher bond strength compared to Adper Single Bond 2; however, its difference from Clearfil Bond U was not significant. Z-Prime Plus, which is light-cured, has a lower viscosity compared to other study groups and so is thinned more easily and its remaining solvent is eliminated then (16,33). In addition, the carboxylic monomer, in association with 10-MDP, helps the chemical reaction of primer with zirconia (11,30), resulting in an increase in the bond strength of zirconia to resin.

 In a study by Amaral et al on the bond strength of resin cement to zirconia with the use of two universal adhesives, Monobond and Scotch Bond Universal, and two zirconia primer groups, Z-Prime Plus and AZ Primer, the highest bond strength was achieved with the use of universal adhesive, consistent with the results of the present study (34). However, in a study by Seabra et al on the shear bond strength of composite resin to zirconia with the use of two universal adhesives, All Bond Universal and Scotch Bond Universal, and Z-Prime, the difference was not significant despite a higher shear bond strength of universal adhesives compared to zirconia primer. Scotch Bond contains silane and as it was explained before, it makes it difficult to eliminate water, resulting in a lower bond strength. In addition, the only functional monomer of All Bond Universal is 10-MDP, and its lower bond strength compared to Futura Bond U (used in present study) might be attributed to the ability of two monomers, i.e. 10-MDP and carboxylic acid to form a chemical bond with zirconia (31,35).

Another reason for the difference in the bond strength of adhesives containing 10-MDP might be the differences in the concentrations of HEMA and water. This volatile resin monomer has a high capacity to wet and penetration into hydrophilic substrates and improves the immediate bond strength of the adhesive systems by a higher penetration into microporosities of the zirconia substrate (16). In addition, it prevents the separation of hydrophilic and hydrophobic phases of the composition through its solvent nature; however, it can not easily be eliminated by air-drying (33). However, HEMA absorbs water in both its polymerized and unpolymerized states and at high concentrations compromises the polymer mechanical properties. In addition, HEMA, in its unpolymerized state, decreases the water vapor pressure and decreases the odds of water evaporation during the water drying stage (16).

Based on the results of the present study, universal adhesives, despite the presence of different functional factors next to each other, were able to form bonds as well as and even better than those with the specific Z-Prime Plus primer with zirconia. The advantage of universal adhesives is their ability to form high-quality bonds with a wide range of dental materials and the ease of the procedural steps. Therefore, it appears these primer-adhesives can be alternatives for specific primers of different substrates. The adhesives that are bonded to zirconia should have a chemical composition with optimal concentrations of compatible hydrophilic and functional agents so that they can boost each other and form a durable and hydrophobic interface after polymerization.

It should be pointed out that use of phosphoric acid for surface preparation of zirconia is contraindicated because the phosphate ion of phosphoric acid is bonded to the surface of zirconia and is not removed even with rinsing with water. In addition, this ion competes with the phosphate ion in the zirconia primer to react with the zirconia surface (16).

However, the manufacture recommends conditioning with phosphoric acid after sandblasting with aluminum oxide particles to apply Clearfil Universal Bond and Adper Single Bond 2 to zirconia substrate, which was the technique used in the present study. Therefore, further studies are necessary to evaluate the effect of shear bond strength of composite resin to zirconia with the use of this adhesive system.

In addition, recent studies have shown that elimination of the solvent with warm air flow and greater pressure is preferable. This approach results in a thinner and more homogeneous primer layer, which is vital for a durable bond between zirconia and the resin (31). Therefore, it is recommended that the bond strength of 10-MDP-contaning primer-adhesives be evaluated by considering factors such as temperature difference and air-drying pressure.

## Conclusions

Based on the results of the preset *in vitro* study, the bond strength of universal adhesive to zirconia was comparable to that of specific primer of zirconia and much higher than that of the adhesive without phosphate ester monomer such as Adper Single Bond 2.
